# Reply: High mortality at safety-net hospitals for patients with cirrhosis

**DOI:** 10.1097/HC9.0000000000000965

**Published:** 2026-06-19

**Authors:** Rebecca G. Kim, Andrea Wallace, Jennifer C. Price

**Affiliations:** 1 Department of Internal Medicine, Division of Gastroenterology and Hepatology, University of Utah School of Medicine, Salt Lake City, Utah, USA; 2 College of Nursing, University of Utah, Salt Lake City, Utah, USA; 3 Department of Medicine, Division of Gastroenterology and Hepatology, University of California San Francisco, San Francisco, California, USA

We appreciate the interest of Shah and colleagues in our article on an acceptable method to incorporate social drivers of health (SDoH) screening into hepatology clinics.^1^ Their multicenter study highlights yet another association between patients’ social context and meaningful clinical outcomes, specifically, the knowledge of documentation status and mortality among patients with cirrhosis cared for in safety-net hospitals. This is an important contribution to the growing body of evidence that SDoH powerfully impact clinical outcomes; however, we believe that addressing their findings in clinical practice first requires a thoughtful, patient-centered assessment.

Patients with chronic liver disease (CLD) who are most negatively impacted by SDoH often experience worse CLD-related outcomes, including increased mortality.^2^ It is important to not only define these relationships but also work toward intervening, as able, to mitigate the role of SDoH on disparate clinical outcomes.

At the University of Utah Health, health-related social needs (HRSN) screening has been incorporated into hepatology clinics, along with other settings.^3^ When HRSN are reported, and if patients are interested, we connect patients to available community resources. The decision to screen for HRSN was intentional; we can identify tangible and modifiable needs for patients. With our screening process, we can act and potentially improve patients’ lives and, over time, their health outcomes.

Another common approach to SDoH screening is capturing social risks, which are measurable, individual-level conditions often shaped by broader structural drivers that increase the likelihood of certain health outcomes.^4^ While some risks may also be intervenable, the changes required are generally at the systems level. Therefore, screening for these data may not lead to a direct, positive change for a patient. Social risk screening has been adopted nationally, though we argue with the addition of certain questions, like documentation status, screening acceptability may suffer. This has been determined by members of our team in pediatrics, where we found Spanish-speaking parents are more likely to respond “prefer not to answer” when offered the option to social needs questions. This approach demonstrates the information we may lose when using check-boxes versus offering to patients what they may or may not wish to disclose; this process is quite vulnerable for patients.^3^^,^^5^

While we agree with Shah et al and advocate for SDoH screening in hepatology practice, we recommend further assessment of patients’ willingness to disclose documentation status, particularly when they may fear serious consequences depending on their response. Indeed, this fear was voiced within our study population when we conducted qualitative interviews asking about patients’ experience with HRSN screening:

It’s kinda, like you said, overwhelming, ‘cause it’s kinda scary for us Spanish people to fill out surveys. ‘Cause we always think somebody’s gonna come and get us because we did drugs or because we’re not from here or just because they want to get into our business. (ID07, female, 57years old)

It is critical to improve access to liver transplantation for the safety-net population; however, we advise a patient-engaged approach to evaluating the best practice to capture documentation status and other social risk factors.
